# Comparison of secular trends of leukemia in China and the United States from 1990 to 2021 and their projections for the next 15 years

**DOI:** 10.3389/fpubh.2024.1425043

**Published:** 2024-08-16

**Authors:** Xinyi Ge, Lifei Zhang, Qiaolei Zhang, Jianhua Feng, Linlin Yang, Yuxin Tong, Shan Zheng, Yamin Tan

**Affiliations:** ^1^Department of Hematology, Zhejiang Cancer Hospital, Hangzhou, China; ^2^Hangzhou Institute of Medicine (HIM), Chinese Academy of Sciences, Hangzhou, China

**Keywords:** leukemia, China, United States, age-period-cohort, secular trends, incidence, mortality

## Abstract

**Background:**

Leukemia imposes a large healthcare burden both in China and the United States (US). The disease burden differs greatly between the two countries, but related research is limited. We explored the differences in leukemia incidence and mortality between China and the US.

**Methods:**

Data on leukemia in China and the US from 1990 to 2021 were collected from the Global Burden of Disease 2021 database. Incidence and mortality were used to estimate the disease burden, and joinpoint regression was performed to compare their secular trends. We used an age-period-cohort model to analyze the effects of age, period, and birth cohort and project future trends in the next 15 years.

**Results:**

In 2021, the age-standardized incidence rate (ASIR) and the age-standardized death rate (ASDR) of leukemia were lower in China than in the US. However, the incidence and mortality of acute lymphoblastic leukemia (ALL) was considerably higher in China. In the past decades, the ASIR showed decreased tendency in the US, while ASIR showed stable in China. The ASDR tended to decrease in both countries from 1990 to 2021. Males have higher rates of incidence and mortality than females in two countries. The age effects showed that children and older individuals have higher RRs for incidence and mortality in China, while the RRs for incidence and mortality in the US particularly increased in the older population. The disease burden of leukemia in children is obviously greater in China. The ASIRs and ASDRs of leukemia will continue to decline in the next 15 years in China and the US, with the US experiencing a more obvious downtrend.

**Conclusions:**

Over the past decades, the ASDRs in two countries both tended to decrease. And compared to the US, China had lower leukemia incidence and mortality, However, the ASIRs in China tended toward stable, which it was showed downtrend in the US. Children have obviously greater RRs for incidence and mortality in China. The incidence and mortality will decrease continuously in two countries. Effective intervention measures are needed to reduce the burden of leukemia.

## Introduction

Leukemias are a group of hematopoietic stem cell malignant clonal diseases (1), including acute lymphoblastic leukemia (ALL), acute myeloid leukemia (AML), chronic lymphocytic leukemia (CLL), chronic myeloid leukemia (CML), and other leukemia according to the Global Burden of Disease Study 2021 (GBD 2021) from Global Health Data Exchange (GHDx). Moreover, hematologic malignancies have been shown to have an increasing disease burden (2), with leukemias having the highest burden among all types (3). According to the GBD 2019, leukemia have affected 643.58 thousand people globally in 2019 (4), and was ranked 7th of the leading causes of disability-adjusted life years (DALYs) among 26 cancers (2). Based on the GLOBOCAN 2020 statistics, 474,519 new diagnoses of leukemia ranked it 15th among 36 cancers (5). The incidence and mortality of this disease vary in different regions. The incidence cases of leukemia was 105,667 in China and 52,060 in the United States (US) in 2021, and 58,903 and 29,785 people died, respectively. Leukemia is life-threatening and has become a serious public health problem in two countries.

With the rapid economic and social development, the cancer spectrum in developing countries is gradually moving toward that of developed countries (6). The strategies and policies of cancer prevention implemented in the US is valuable for China to establish an effective cancer control system which is more in line with China's current conditions. Differences in all cancers between China and the US have been reported before (7, 8), but no studies have focused on leukemia. Therefore, in this study, we compare and explore the disease characteristics and development trends of leukemia between China and the US. Based on GBD 2021, we investigated secular trends in leukemia incidence and mortality from 1990 to 2021. We used an age–period–cohort (APC) model to reveal differences in leukemia among different age groups, periods groups, birth cohorts between China and the US. A Bayesian age–period–cohort (BAPC) model was applied to predict the trend of leukemia incidence and mortality over the next 15 years.

## Materials and methods

### Study data

GBD 2021 synthesized a large number of data input sources including surveys, censuses, vital statistics, and other health-related data sources and then quantified health loss for 371 diseases in 204 countries and territories, including measures of incidence and death that together constitute a comprehensive assessment of disease burden (9). And the methodology for the estimation models has been described elsewhere before (10, 11). We extracted information about leukemia in China and the US between 1990 and 2021 from the Global Health Data Exchange (GHDx) query tool (http://ghdx.healthdata.org/gbd-results-tool). We chose “China” and “United States of America” as locations and leukemia and its subtypes as causes, incidences and deaths as measures. We also collected information on 20 age groups, which were divided into 5 years from 0 to 95+ years, and data for each sex of leukemia patients were collected. We used age-standardized rates (ASRs), the absolute incidence and mortality to analyze one aspect of differences between two countries. The ASRs and 95% uncertainty intervals (UIs) were calculated on the basis of the GBD 2021 global age-standard population (12, 13).

### Joinpoint regression analysis

Kim proposed the Joinpoint regression model for the first time in 1998 (14). The core idea of this model is to establish segmented regression according to the time characteristics of disease distribution, divide the research time into different intervals through several connection points, and carry out trend fitting and optimization for each interval. Furthermore, the disease characteristics specific to different intervals in the global time range were evaluated in more detail. We calculated the average annual percent change (AAPC) and annual percent change (APC) in this research. When the AAPC/APC ratio and the lower limit of the 95% confidence interval (CI) are both positive, an upward trend is indicated. Conversely, when the AAPC/APC ratio and the upper limit of the 95% CI are both negative, there is a downward trend. Joinpoint regression 4.9 software (National Cancer Institute, Rockville, MD, USA) was used.

### Age-period-cohort analysis

Due to the obvious collinearity of age, period and birth cohort, it is difficult to determine the independent influence of these three factors (15, 16). The Age-period-cohort model decomposes the time dimension into age effect, period effect and birth cohort effect (17). Age effects are changes related to the biological and social processes of aging that are specific to the individual, which including physiological changes and the accumulation of social experiences associated with aging, but are independent of the time period or birth cohort. The period effect is the result of external factors affecting all age groups equally at a particular calendar time, which could result from a range of environmental, social and economic factors, such as war, famine and economic crisis. Birth cohort effects are the changes that result from a unique exposure of a cohort over time, which is the sum of all unique exposures experienced by the cohort from birth. To figure out the independent effects of age, period, and birth cohort, we applied the intrinsic estimation (IE) method, which has been proven to solve this problem (18, 19). To satisfy the framework of the model, we extracted the rates from GBD data and appropriately divided them into successive 5-year groups (< 5, 5–9... 95+ years), consecutive 5-year periods from 1990 to 2021, and corresponding consecutive 5-year birth cohort groups starting from 1897–1901 to 2017–2021. The estimated coefficients for the age, period and birth cohort effects can be calculated via the IE method. These coefficients were transformed into exponential values [exp(coef.) = e^coef.^], which denotes the relative risk (RR) of a particular age, period or birth cohort relative to the average level of all ages, periods or birth cohorts combined (12, 16). For example, the RR of leukemia incidence in females aged 0–4 years was 4.44, indicating that the risk in this age group was 4.44 times greater than that in all other age groups (18). The APC IE model was established by Stata MP 15 (64-bit) software (StataCorp LP, TX, USA).

### Bayesian age–period–cohort analysis

Bayesian age–period–cohort (BAPC) model was widely applied to predict disease trends in populations in the literature before (20–23), which has obtained well-calibrated probabilistic forecasts and the range is not too wide (20, 23, 24). The formula has been described in previous reports (21, 22). We predicted the ASRs of leukemia incidence and death in China and the US from 2022 to 2036 by BAPC analysis which was performed with BAPC package (version 0.0.36) and integrated nested Laplace approximation (INLA) package (version 22.05.07). All data analyses were conducted by R program (version 4.2.0, Bell Laboratories, Murray Hill, NJ, USA).

## Results

### Descriptive analysis

**Figure 1:**
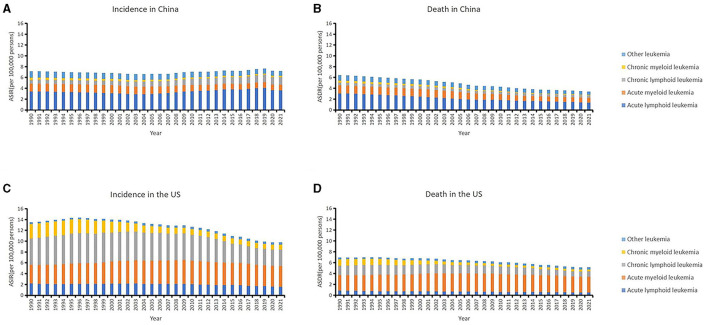
The trends of country-specific age-standardized incidence rates (ASIRs), age-standardized death rates (ASDRs) of leukemia and its subtypes from 1990 to 2021 in China **(A, B)** and in the US **(C, D)**.

The tendencies of leukemia and its subtypes in China and the US over the past decades are shown in Figure 1 and Tables 1, 2. Among the leukemia subtypes, ALL dominated in China, and AML dominated in the US.

**Table 1:** The age-standardized incidence rates (ASIRs) and age-standardized death rates (ASDRs) of leukemia in China and the US in 1990 and 2021 and their annual percentage changes from 1990 to 2021.

		**China**				**United States of America**			
		**Rates in 1990, 95% UI**	**Rates in 2021, 95% UI**	***P*-value**	**AAPC_95CI**	**Rates in 1990, 95% UI**	**Rates in 2021, 95% UI**	***P*-value**	**AAPC_95CI**
**ASIRs**									
**Leukemia**									
Both		7.14 (5.52, 8.58)	7.21 (4.93, 9.05)	0.884	−0.02 (−0.22, 0.19)	13.50 (12.91, 13.84)	9.81 (9.11, 10.22)	< 0.001	−1.04 (−1.2, −0.87)
Female		6.68 (4.59, 8.24)	5.92 (3.44, 7.70)	< 0.001	−0.44 (−0.69, −0.2)	10.02 (9.40, 10.38)	7.53 (6.82, 7.93)	< 0.001	−0.96 (−1.22, −0.69)
Male		7.71 (5.07, 9.99)	8.51 (5.14, 11.38)	0.004	0.27 (0.08, 0.45)	18.03 (17.42, 18.49)	12.56 (11.73, 13.07)	< 0.001	−1.13 (−1.31, −0.96)
	**ALL**								
	Both	3.38 (2.39, 4.49)	3.64 (2.00, 5.05)	0.695	0.09 (−0.35, 0.53)	2.19 (2.11, 2.28)	1.58 (1.47, 1.70)	< 0.001	−0.84 (−1.02, −0.66)
	Female	3.36 (2.03, 4.69)	3.03 (1.29, 4.37)	0.02	−0.49 (−0.91, −0.08)	1.89 (1.78, 2.01)	1.45 (1.33, 1.58)	< 0.001	−0.72 (−0.98, −0.46)
	Male	3.44 (1.85, 5.03)	4.20 (1.92, 6.08)	0.008	0.49 (0.13, 0.86)	2.51 (2.39, 2.62)	1.72 (1.59, 1.86)	< 0.001	−1.18 (−1.82, −0.54)
	**AML**								
	Both	1.46 (0.80, 2.24)	1.03 (0.69, 1.45)	< 0.001	−1.1 (−1.22, −0.97)	3.46 (3.28, 3.55)	3.85 (3.53, 4.01)	< 0.001	0.33 (0.21, 0.45)
	Female	1.34 (0.70, 1.99)	0.91 (0.59, 1.47)	< 0.001	−1.18 (−1.39, −0.97)	2.92 (2.73, 3.02)	3.14 (2.84, 3.31)	< 0.001	0.26 (0.14, 0.38)
	Male	1.61 (0.69, 2.71)	1.17 (0.60, 1.78)	< 0.001	−1.04 (−1.18, −0.89)	4.23 (4.07, 4.34)	4.73 (4.41, 4.93)	0.015	0.3 (0.06, 0.54)
	**CLL**								
	Both	0.72 (0.42, 0.98)	1.42 (0.88, 2.00)	< 0.001	2.25 (2.15, 2.35)	4.87 (4.57, 5.04)	3.04 (2.76, 3.22)	< 0.001	−1.57 (−1.92, −1.22)
	Female	0.63 (0.32, 0.95)	1.15 (0.48, 1.66)	< 0.001	2.02 (1.86, 2.19)	3.53 (3.24, 3.70)	2.17 (1.88, 2.31)	< 0.001	−1.76 (−2.49, −1.04)
	Male	0.83 (0.39, 1.20)	1.71 (0.87, 2.69)	< 0.001	2.39 (2.3, 2.48)	6.69 (6.37, 6.92)	4.12 (3.82, 4.35)	< 0.001	−1.57 (−1.87, −1.27)
	**CML**								
	Both	0.38 (0.17, 0.55)	0.21 (0.11, 0.33)	< 0.001	−1.83 (−2.18, −1.48)	2.63 (2.52, 2.73)	0.88 (0.83, 0.93)	< 0.001	−3.53 (−3.67, −3.39)
	Female	0.37 (0.15, 0.62)	0.18 (0.10, 0.28)	< 0.001	−2.33 (−2.58, −2.08)	1.44 (1.35, 1.52)	0.50 (0.46, 0.54)	< 0.001	−3.4 (−3.64, −3.16)
	Male	0.39 (0.09, 0.59)	0.25 (0.06, 0.44)	< 0.001	−1.41 (−1.77, −1.05)	4.11 (3.95, 4.27)	1.33 (1.25, 1.39)	< 0.001	−3.63 (−3.76, −3.5)
	**Other leukemia**								
	Both	1.20 (0.70, 1.66)	0.91 (0.44, 1.26)	< 0.001	−0.88 (−1.1, −0.67)	0.34 (0.32, 0.36)	0.46 (0.41, 0.48)	< 0.001	1.01 (0.75, 1.26)
	Female	0.98 (0.48, 1.51)	0.65 (0.24, 1.06)	< 0.001	−1.25 (−1.62, −0.89)	0.24 (0.22, 0.26)	0.28 (0.25, 0.31)	< 0.001	0.48 (0.29, 0.67)
	Male	1.44 (0.67, 2.38)	1.18 (0.49, 1.89)	< 0.001	−0.64 (−0.76, −0.52)	0.49 (0.46, 0.51)	0.67 (0.61, 0.70)	< 0.001	1.14 (0.82, 1.45)
**ASDRs**									
**Leukemia**									
Both		6.46 (5.04, 7.66)	3.42 (2.51, 4.26)	< 0.001	−2.03 (−2.16, −1.89)	6.97 (6.59, 7.18)	5.17 (4.69, 5.41)	< 0.001	−1.04 (−1.14, −0.94)
Female		5.97 (4.19, 7.39)	2.70 (1.77, 3.50)	< 0.001	−2.52 (−2.68, −2.37)	5.40 (5.00, 5.61)	3.90 (3.46, 4.13)	< 0.001	−1.11 (−1.2, −1.01)
Male		7.08 (4.71, 9.27)	4.22 (2.73, 5.71)	< 0.001	−1.67 (−1.78, −1.56)	9.26 (8.86, 9.50)	6.79 (6.26, 7.09)	< 0.001	−1.03 (−1.15, −0.91)
	**ALL**								
	Both	3.05 (2.17, 4.02)	1.36 (0.78, 1.75)	< 0.001	−2.61 (−2.83, −2.38)	0.88 (0.86, 0.89)	0.51 (0.49, 0.53)	< 0.001	−1.65 (−1.94, −1.36)
	Female	2.97 (1.81, 4.12)	1.06 (0.45, 1.40)	< 0.001	−3.31 (−3.54, −3.07)	0.72 (0.69, 0.74)	0.43 (0.41, 0.45)	< 0.001	−1.64 (−1.84, −1.44)
	Male	3.16 (1.75, 4.64)	1.65 (0.77, 2.33)	< 0.001	−2.08 (−2.25, −1.9)	1.06 (1.04, 1.08)	0.60 (0.57, 0.62)	< 0.001	−1.74 (−2.24, −1.24)
	**AML**								
	Both	1.45 (0.80, 2.21)	0.88 (0.59, 1.24)	< 0.001	−1.59 (−1.77, −1.41)	2.86 (2.70, 2.94)	2.91 (2.66, 3.03)	0.792	0.02 (−0.15, 0.2)
	Female	1.31 (0.69, 1.94)	0.74 (0.49, 1.22)	< 0.001	−1.77 (−1.98, −1.56)	2.36 (2.19, 2.45)	2.27 (2.04, 2.39)	0.175	−0.13 (−0.31, 0.06)
	Male	1.62 (0.70, 2.67)	1.03 (0.55, 1.59)	< 0.001	−1.45 (−1.61, −1.29)	3.59 (3.45, 3.69)	3.72 (3.46, 3.88)	0.569	0.06 (−0.15, 0.27)
	**CLL**								
	Both	0.55 (0.33, 0.74)	0.44 (0.28, 0.63)	< 0.001	−0.72 (−0.9, −0.54)	1.72 (1.58, 1.80)	1.00 (0.87, 1.07)	< 0.001	−1.79 (−2.14, −1.45)
	Female	0.45 (0.23, 0.66)	0.31 (0.13, 0.45)	< 0.001	−1.18 (−1.51, −0.85)	1.16 (1.02, 1.23)	0.67 (0.55, 0.73)	< 0.001	−1.93 (−2.4, −1.46)
	Male	0.68 (0.33, 0.97)	0.60 (0.33, 0.93)	< 0.001	−0.43 (−0.64, −0.22)	2.58 (2.41, 2.68)	1.45 (1.29, 1.54)	< 0.001	−1.85 (−2.18, −1.52)
	**CML**								
	Both	0.36 (0.17, 0.52)	0.10 (0.06, 0.16)	< 0.001	−4.07 (−4.35, −3.8)	1.17 (1.11, 1.22)	0.32 (0.29, 0.34)	< 0.001	−4.14 (−4.38, −3.91)
	Female	0.35 (0.15, 0.58)	0.08 (0.05, 0.14)	< 0.001	−4.63 (−4.91, −4.35)	0.90 (0.84, 0.95)	0.25 (0.22, 0.27)	< 0.001	−4.13 (−4.45, −3.82)
	Male	0.37 (0.08, 0.57)	0.12 (0.03, 0.22)	< 0.001	−3.58 (−3.98, −3.18)	1.54 (1.48, 1.60)	0.41 (0.37, 0.43)	< 0.001	−4.25 (−4.45, −4.04)
	**Other leukemia**								
	Both	1.05 (0.64, 1.49)	0.65 (0.34, 0.88)	< 0.001	−1.55 (−1.78, −1.33)	0.35 (0.32, 0.37)	0.43 (0.39, 0.46)	< 0.001	0.71 (0.51, 0.91)
	Female	0.89 (0.44, 1.41)	0.50 (0.20, 0.81)	< 0.001	−1.81 (−2.1, −1.51)	0.26 (0.23, 0.28)	0.28 (0.24, 0.31)	0.005	0.33 (0.1, 0.56)
	Male	1.25 (0.58, 2.04)	0.82 (0.37, 1.30)	< 0.001	−1.37 (−1.57, −1.18)	0.49 (0.46, 0.52)	0.62 (0.56, 0.66)	< 0.001	0.88 (0.59, 1.18)

ALL, acute lymphoblastic leukemia; AML, acute myeloid leukemia; CLL, chronic lymphocytic leukemia; CML, chronic myeloid leukemia; UI, uncertainty interval; CI, confidence interval; AAPC, average annual percent change; ASIRs, age–standardized incidence rates; ASDRs, age–standardized death rates.

**Table 2:** The rates of leukemia in different age group in 2021 and their percentage changes from 1990 to 2021 in China and the US.

**Categories**	**China**		**United States of America**	
	**Rates in 2019, 95% UI**	**AAPC_95CI**	**Rates in 2019, 95% UI**	**AAPC_95CI**
**Incidence**				
< 5	16.31 (8.40, 25.82)	−0.22 (−0.76, 0.32)	7.14 (6.24, 8.22)	−0.67 (−0.97, −0.37)
5–9	4.75 (2.98, 6.43)	−0.36 (−0.83,0.11)	2.14 (1.94, 2.39)	−1.71 (−2.63, −0.80)
10–14	3.60 (2.49, 4.50)	−0.64 (−1.11,−0.17)	1.93 (1.77, 2.10)	−1.30 (−1.70, −0.91)
15–19	3.49 (2.28, 4.48)	−0.52 (−0.75,−0.29)	1.64 (1.52, 1.77)	−1.71 (−2.14, −1.28)
20–24	3.73 (2.49, 4.69)	0.56 (0.38, 0.74)	1.82 (1.72, 1.93)	−0.97 (−1.52, −0.42)
25–29	3.43 (2.28, 4.27)	0.56 (0.13, 0.99)	1.95 (1.87, 2.05)	−1.24 (−1.7, −0.78)
30–34	3.47 (2.42, 4.33)	0.03 (−0.11, 0.17)	1.98 (1.89, 2.08)	−1.74 (−2.1, −1.37)
35–39	4.13 (2.92, 5.19)	−0.44 (−0.61, −0.27)	2.79 (2.66, 2.90)	−1.68 (−1.99, −1.37)
40–44	5.15 (3.72, 6.63)	−0.2 (−0.53, 0.14)	3.59 (3.42, 3.76)	−1.90 (−2.13, −1.67)
45–49	5.46 (3.89, 7.33)	−0.35 (−0.79, 0.09)	5.20 (5.01, 5.44)	−2.03 (−2.44, −1.61)
50–54	7.28 (5.02, 9.76)	−0.06 (−0.22, 0.09)	7.91 (7.63, 8.18)	−2.21 (−2.88, −1.53)
55–59	8.90 (6.28, 11.87)	−0.17 (−0.44, 0.09)	13.27 (12.88, 13.68)	−2.00 (−2.35, −1.64)
60–64	11.57 (8.10, 15.09)	0.21 (0.06, 0.35)	20.80 (20.02, 21.50)	−1.89 (−2.23, −1.55)
65–69	13.22 (9.19, 17.16)	0.18 (−0.16, 0.52)	32.29 (30.58, 33.49)	−1.54 (−1.89, −1.19)
70–74	16.71 (11.84, 21.37)	0.27 (0.13, 0.41)	52.17 (48.47, 54.30)	−1.02 (−1.29, −0.75)
75–79	18.72 (14.05, 23.79)	0.17 (0.02, 0.33)	80.08 (72.37, 83.88)	−0.52 (−0.78, −0.25)
80–84	15.83 (12.07, 20.76)	−0.11 (−0.31, 0.10)	96.49 (80.67, 104.49)	0.17 (0.03, 0.31)
85–89	18.57 (14.13, 24.54)	−0.01 (−0.39, 0.37)	127.58 (101.04, 140.15)	0.55 (0.41, 0.68)
90–94	16.59 (12.77, 21.67)	−0.44 (−0.54, −0.35)	146.41 (110.68, 165.70)	0.78 (0.60, 0.97)
95 plus	11.51 (8.34, 14.81)	−0.18 (−0.36, −0.01)	117.07 (83.56, 135.11)	0.46 (−0.02, 0.94)
**Deaths**				
< 5	1.67 (1.00, 2.50)	−6.46 (−6.78, −6.14)	0.62 (0.55, 0.69)	−2.12 (−2.63, −1.60)
5–9	1.65 (1.10, 2.18)	−3.54 (−3.8, −3.27)	0.50 (0.48, 0.54)	−2.94 (−3.58, −2.30)
10–14	1.58 (1.18, 1.95)	−3.09 (−3.63, −2.54)	0.60 (0.57, 0.63)	−2.52 (−2.83, −2.20)
15–19	1.99 (1.36, 2.53)	−2.22 (−2.37, −2.08)	0.67 (0.63, 0.71)	−2.79 (−3.15, −2.43)
20–24	2.24 (1.54, 2.80)	−0.99 (−1.22, −0.76)	0.91 (0.87, 0.95)	−1.83 (−2.01, −1.65)
25–29	1.95 (1.34, 2.44)	−0.89 (−1.17, −0.62)	0.96 (0.93, 1.00)	−1.73 (−2.38, −1.07)
30–34	2.11 (1.50, 2.63)	−1.25 (−2.03, −0.47)	0.99 (0.95, 1.04)	−1.99 (−2.39, −1.59)
35–39	2.39 (1.72, 3.02)	−1.57 (−1.73, −1.41)	1.34 (1.28, 1.39)	−1.73 (−1.94, −1.52)
40–44	2.69 (1.96, 3.45)	−1.83 (−2.08, −1.57)	1.72 (1.65, 1.79)	−2.04 (−2.13, −1.95)
45–49	2.83 (2.07, 3.73)	−1.94 (−2.52, −1.36)	2.45 (2.37, 2.54)	−1.97 (−2.07, −1.88)
50–54	3.69 (2.67, 4.76)	−1.77 (−1.92, −1.63)	3.61 (3.50, 3.73)	−2.02 (−2.40, −1.65)
55–59	4.92 (3.58, 6.33)	−1.79 (−1.91, −1.66)	6.10 (5.94, 6.26)	−1.88 (−2.00, −1.75)
60–64	6.84 (4.90, 8.71)	−1.25 (−1.49, −10)	9.93 (9.58, 10.22)	−1.71 (−1.86, −1.56)
65–69	8.90 (6.39, 11.35)	−1.07 (−1.35, −0.80)	16.26 (15.41, 16.83)	−1.43 (−1.65, −1.20)
70–74	12.33 (8.93, 15.61)	−0.85 (−0.99, −0.70)	27.96 (26.15, 29.06)	−0.97 (−1.17, −0.77)
75–79	15.11 (10.99, 19.43)	−0.86 (−1.07, −0.65)	45.36 (41.21, 47.42)	−0.51 (−0.73, −0.29)
80–84	16.32 (12.49, 21.10)	−0.73 (−0.95, −0.51)	68.52 (58.21, 73.99)	−0.10 (−0.21, 0.02)
85–89	20.81 (16.20, 27.24)	−0.62 (−0.94, −0.29)	96.63 (77.40, 105.82)	0.21 (−0.06, 0.48)
90–94	20.28 (15.65, 27.08)	−0.98 (−1.09, −0.87)	122.80 (93.63, 138.01)	0.35 (0.12, 0.58)
95 plus	16.32 (11.74, 20.93)	−0.57 (−0.77, −0.38)	137.73 (97.85, 158.43)	0.48 (0.10, 0.86)

UI, uncertainty interval; CI, confidence interval; AAPC, average annual percent change.

In China, the ASIR remain stable on the whole which was 7.14 (95% UI: 5.52, 8.58) in 1990 and 7.21 (95% UI: 4.93, 9.05) in 2021. The ASDR decreased from 6.46 (95% UI: 5.04, 7.66) in 1990 to 3.42 (95% UI: 2.51, 4.26) in 2021 (Table 1). Patients aged 75–79 years had the highest incidence rate (18.72, 95% UI: 14.05, 23.79) and patients aged 85–89 years had the highest death rate (20.81, 95% UI: 16.20, 27.24). In addition, the 0–4 years age group also had a high incidence rate (16.31, 95% UI: 8.40, 25.82; Table 2).

In the US, the ASIR of leukemia decreased from 13.50 (95% UI: 12.91, 13.84) in 1990 to 9.81 (95% UI: 9.11, 10.22) in 2021. The ASDR decreased from 6.97 (95% UI: 6.59, 7.18) in 1990 to 5.17 (95% UI: 4.69, 5.41) in 2021 (Table 1). The age group among 90–94 years had the highest incidence rate (146.41, 95% UI: 110.68, 165.70) and age group older than 95 years had the highest death rate (137.73, 95% UI: 97.85, 158.43; Table 2).

The US had a greater burden of leukemia than did in China. The ASIR of ALL has remained high in the past decades and was much higher than in the US. The burdens of AML, CLL, and CML were greater in the US. Furthermore, the analysis revealed that males have greater burdens of ASIRs and ASDRs than females in any subtypes, whether in China or the US.

### Joinpoint regression analysis

**Figure 2:**
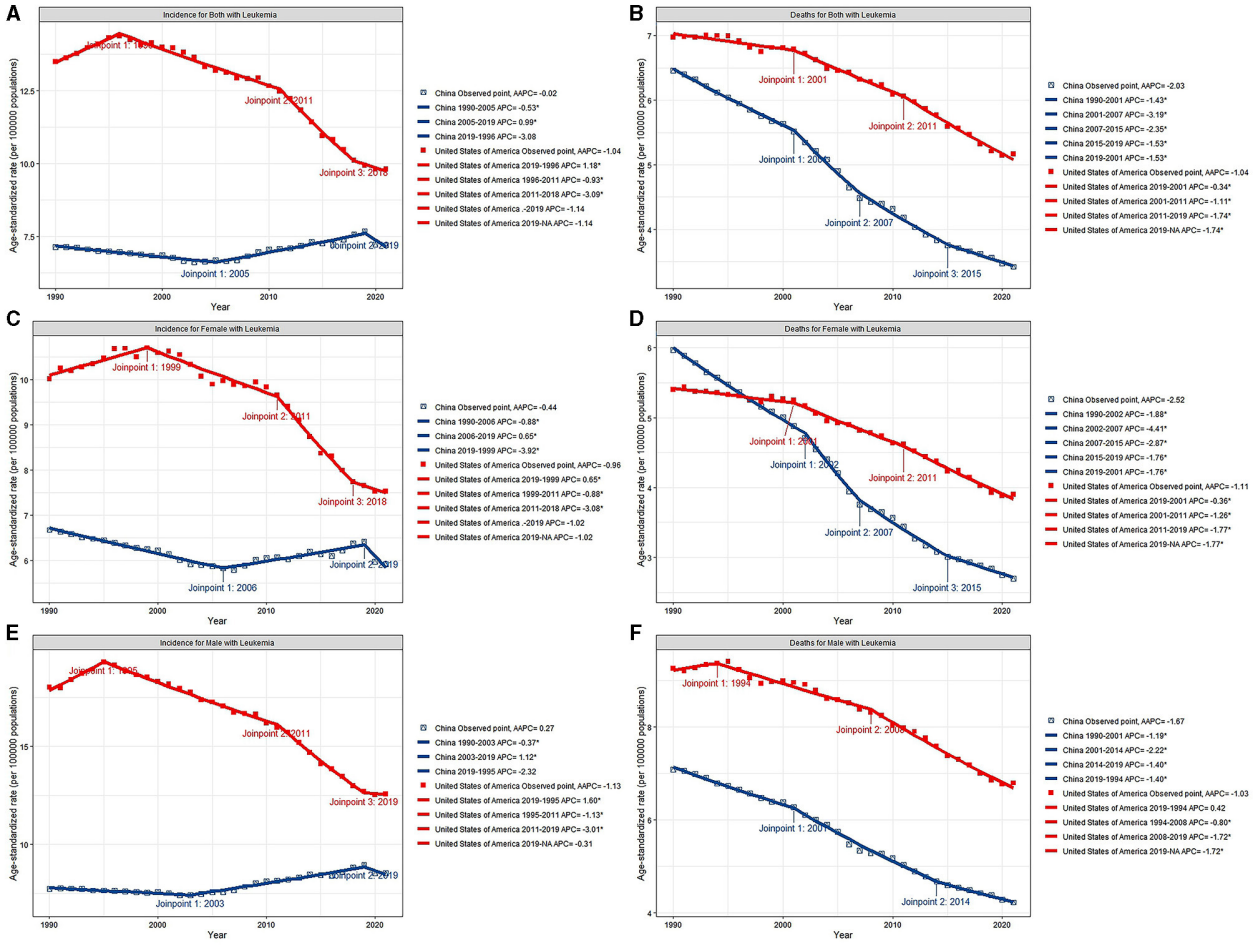
Joinpoint regression analysis of ASIR for both sex **(A)**, female **(C)** and male **(E)**, ASDR for both sex **(B)**, female **(D)** and male **(F)** of leukemia from 1990 to 2021 in China and the US. ^*^p < 0.05.

The AAPCs in the ASIRs and ASDRs of the two countries are listed in Figure 2 and Tables 1, 2.

In China, the AAPCs in the ASIR and ASDR for both sexes were −0.02 (95% CI: −0.22, 0.19) and −2.03 (95% CI: −2.16, −1.89), respectively. After a continuous decline, the ASIRs of leukemia have been on the rise since 2005, with a 2005–2019 APC of 0.99 (95% CI: 0.86, 1.12; Figure 2A). Further study revealed that male sex plays a vital role in increasing the incidence of this disease. Among the subtypes, only CLL subtypes showed a sharp upward trend in the annual change in the ASIR over the past 32 years (AAPC: 2.25, 95% CI: 2.15, 2.35). Other types, namely, AML, CML, and other leukemia, all showed declined trends, with AAPCs of −1.1 (95% CI: −1.22, −0.97), −1.83 (95% CI: −2.18, −1.48), and −0.88 (95% CI: −1.1, −0.67), respectively. And ALL remained stable with a AAPC of 0.09 (95% CI: −0.35, 0.53). The ASDRs declined in any subtypes, with a AAPC of −2.61 (95% CI: −2.83, −2.38), −1.59 (95% CI: −1.77, −1.41), −0.72 (95% CI: −0.9, −0.54), −4.07 (95% CI: −4.35, −3.8), and −1.55 (95% CI: −1.78, −1.33) in ALL, AML, CLL, CML, and other leukemia, respectively (Table 1). Patients aged < 5 years showed distinct decreases in death, with AAPC of −6.46 (95% CI: −6.78, −6.14; Table 2).

In the US, the AAPCs in the ASIR and ASDR for both sexes were −1.04 (95% CI: −1.2, −0.87) and −1.04 (95% CI: −1.14, −0.94), respectively. The decline of ASDRs of leukemia patients has accelerated since 2011 (Figure 2B), with a 2011–2021 APC of −1.74 (95% CI: −1.96, −1.53). Among the subtypes, the ASIR increased by 0.33 (95% CI: 0.21, 0.45) and 1.01 (95% CI: 0.75, 1.26) annually in AML and other leukemia. For the ASDR, AML remained stable (AAPC: 0.02, 95% CI: −0.15, 0.2) and other leukemia showed upward (AAPC: 0.71, 95% CI: 0.51, 0.91). The ASIRs and ASDRs of ALL, CLL and CML subtypes all tended to decrease (Table 1). Over the past 30 years, morbidity and mortality have increased in general among people over the age of 80 (Table 2).

The ASDRs of leukemia decreased in both male and female in the two countries, while the annual change was greater in China. Even so, The ASIR of China maintained stable rather than declining. In addition, the annual change for ASDR was more obvious in females than in males in both China and the US.

### Age–period–cohort analysis with the intrinsic estimator

**Figure 3:**
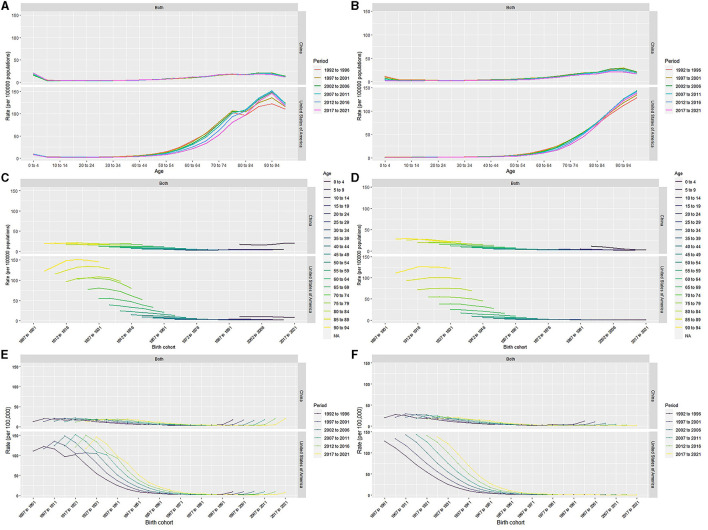
Age-period-specific incidence rates, mortality rates of leukemia for total population in China and the US **(A, B)**. Age-cohort-specific incidence rates, mortality rates of leukemia for total population in China and the US **(C, D)**. Period-cohort-specific incidence rates, mortality rates of leukemia for total population in China and the US **(E, F)**.

**Figure 4:**
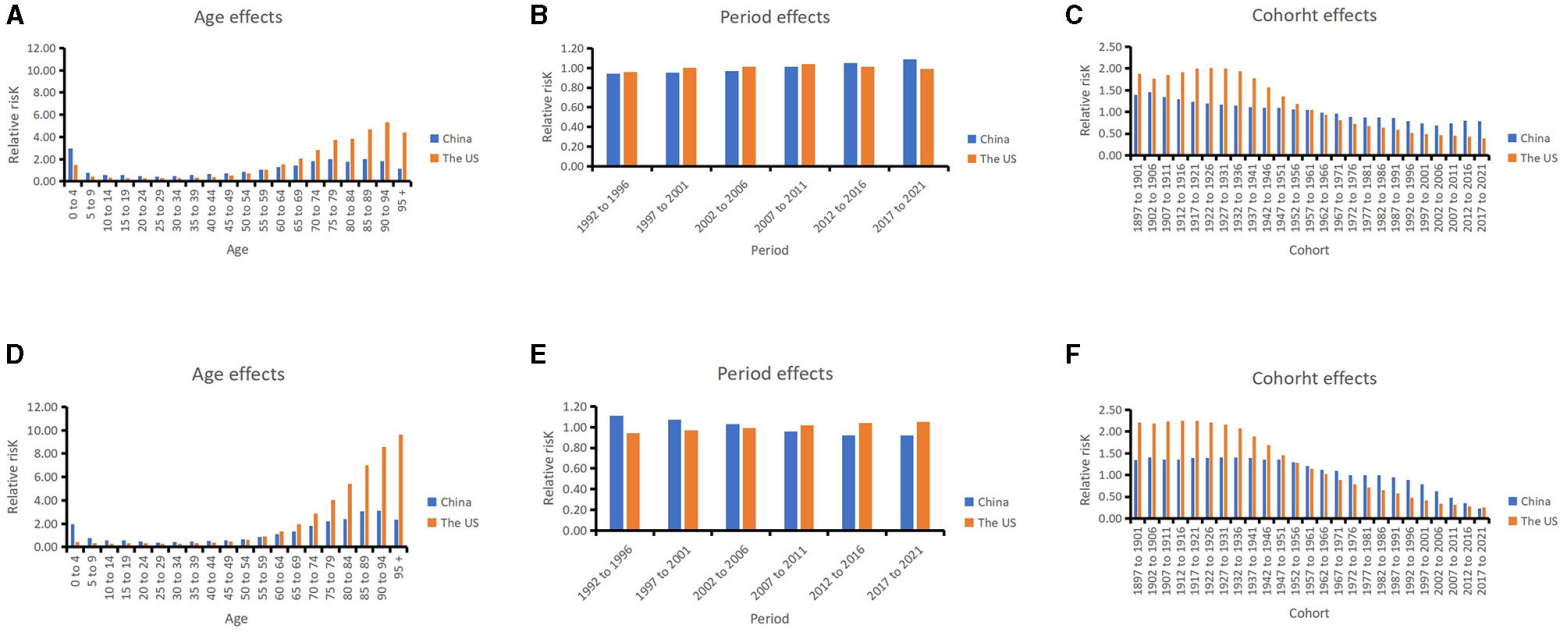
Relative risks of leukemia incidence due to age **(A)**, period **(B)**, and cohort **(C)** effects in China and US from 1992 to 2021; relative risks of leukemia death due to age **(D)**, period **(E)**, and cohort **(F)** effects in China and US from 1992 to 2021.

The pairwise relationships between age, period and cohort are shown in Figure 3. The RRs of leukemia due to age, period, and cohort effects in China and the US are listed in Figure 4 and Table 3.

**Table 3:** Relative risks of leukemia due to age, period and cohort effects in China and the US from 1992 to 2021.

**Categories**	**China**				**United States of America**			
	**Incidence**		**Mortality**		**Incidence**		**Mortality**	
	**RR (95% CI)**	***P*** **-value**	**RR (95% CI)**	***P*** **-value**	**RR (95% CI)**	***P*** **-value**	**RR (95% CI)**	***P*** **-value**
**Age effects**								
0–4	2.99 (2.84, 3.16)	< 0.001	1.95 (1.87, 2.04)	< 0.001	1.46 (1.42, 1.50)	< 0.001	0.41 (0.38, 0.43)	< 0.001
5–9	0.77 (0.73, 0.81)	< 0.001	0.76 (0.73, 0.79)	< 0.001	0.44 (0.43, 0.46)	< 0.001	0.31 (0.29, 0.33)	< 0.001
10–14	0.58 (0.56, 0.61)	< 0.001	0.56 (0.54, 0.58)	< 0.001	0.34 (0.33, 0.36)	< 0.001	0.30 (0.28, 0.32)	< 0.001
15–19	0.56 (0.54, 0.58)	< 0.001	0.56 (0.54, 0.58)	< 0.001	0.29 (0.28, 0.30)	< 0.001	0.31 (0.30, 0.33)	< 0.001
20–24	0.50 (0.48, 0.52)	< 0.001	0.47 (0.46, 0.49)	< 0.001	0.28 (0.27, 0.29)	< 0.001	0.33 (0.31, 0.35)	< 0.001
25–29	0.45 (0.43, 0.46)	< 0.001	0.38 (0.37, 0.39)	< 0.001	0.27 (0.26, 0.28)	< 0.001	0.30 (0.28, 0.31)	< 0.001
30–34	0.47 (0.45, 0.48)	< 0.001	0.41 (0.40, 0.42)	< 0.001	0.27 (0.26, 0.28)	< 0.001	0.29 (0.27, 0.30)	< 0.001
35–39	0.59 (0.58, 0.60)	< 0.001	0.50 (0.49, 0.50)	< 0.001	0.33 (0.32, 0.34)	< 0.001	0.32 (0.30, 0.33)	< 0.001
40–44	0.66 (0.65, 0.67)	< 0.001	0.54 (0.53, 0.55)	< 0.001	0.40 (0.39, 0.41)	< 0.001	0.38 (0.37, 0.40)	< 0.001
45–49	0.70 (0.69, 0.71)	< 0.001	0.56 (0.55, 0.57)	< 0.001	0.53 (0.51, 0.54)	< 0.001	0.48 (0.46, 0.49)	< 0.001
50–54	0.86 (0.85, 0.87)	< 0.001	0.68 (0.66, 0.69)	< 0.001	0.73 (0.72, 0.75)	< 0.001	0.63 (0.61, 0.65)	< 0.001
55–59	1.06 (1.04, 1.08)	< 0.001	0.87 (0.85, 0.89)	< 0.001	1.07 (1.05, 1.09)	< 0.001	0.90 (0.88, 0.93)	< 0.001
60–64	1.28 (1.25, 1.31)	< 0.001	1.09 (1.07, 1.12)	< 0.001	1.52 (1.49, 1.54)	< 0.001	1.34 (1.31, 1.38)	< 0.001
65–69	1.45 (1.41, 1.49)	< 0.001	1.36 (1.33, 1.39)	< 0.001	2.06 (2.03, 2.09)	< 0.001	1.94 (1.90, 1.98)	< 0.001
70–74	1.80 (1.74, 1.85)	< 0.001	1.81 (1.76, 1.86)	< 0.001	2.84 (2.81, 2.88)	< 0.001	2.87 (2.82, 2.92)	< 0.001
75–79	2.02 (1.94, 2.09)	< 0.001	2.21 (2.14, 2.27)	< 0.001	3.73 (3.69, 3.78)	< 0.001	4.00 (3.93, 4.07)	< 0.001
80–84	1.77 (1.70, 1.85)	< 0.001	2.39 (2.31, 2.48)	< 0.001	3.82 (3.77, 3.88)	< 0.001	5.39 (5.29, 5.49)	< 0.001
85–89	2.03 (1.92, 2.13)	< 0.001	3.07 (2.94, 3.20)	< 0.001	4.70 (4.63, 4.78)	< 0.001	6.97 (6.83, 7.12)	< 0.001
90–94	1.84 (1.72, 1.97)	< 0.001	3.12 (2.95, 3.31)	< 0.001	5.30 (5.18, 5.41)	< 0.001	8.55 (8.32, 8.78)	< 0.001
95 +	1.17 (1.01, 1.37)	0.039	2.35 (2.08, 2.67)	< 0.001	4.42 (4.26, 4.59)	< 0.001	9.61 (9.24, 9.99)	< 0.001
**Period effects**								
1992–1996	0.94 (0.93, 0.95)	< 0.001	1.11 (1.10, 1.13)	< 0.001	0.96 (0.95, 0.97)	< 0.001	0.94 (0.93, 0.95)	< 0.001
1997–2001	0.95 (0.94, 0.96)	< 0.001	1.07 (1.06, 1.09)	< 0.001	1.00 (0.99, 1.01)	0.946	0.97 (0.96, 0.98)	< 0.001
2002–2006	0.97 (0.96, 0.97)	< 0.001	1.03 (1.02, 1.03)	< 0.001	1.01 (1.00, 1.02)	0.021	0.99 (0.98, 1.01)	0.302
2007–2011	1.01 (1.01, 1.02)	0.001	0.96 (0.95, 0.97)	< 0.001	1.04 (1.03, 1.04)	< 0.001	1.02 (1.01, 1.03)	0.003
2012–2016	1.05 (1.04, 1.06)	< 0.001	0.92 (0.91, 0.93)	< 0.001	1.01 (1.00, 1.02)	0.003	1.04 (1.02, 1.05)	< 0.001
2017–2021	1.09 (1.07, 1.11)	< 0.001	0.92 (0.91, 0.93)	< 0.001	0.99 (0.98, 1.00)	0.005	1.05 (1.04, 1.06)	< 0.001
**Cohort effects**								
1897–1901	1.39 (0.68, 2.85)	0.370	1.34 (0.77, 2.34)	0.307	1.87 (1.69, 2.08)	< 0.001	2.21 (2.00, 2.45)	< 0.001
1902–1906	1.45 (1.18, 1.78)	< 0.001	1.40 (1.18, 1.66)	< 0.001	1.76 (1.67, 1.85)	< 0.001	2.19 (2.08, 2.32)	< 0.001
1907–1911	1.34 (1.19, 1.50)	< 0.001	1.36 (1.24, 1.50)	< 0.001	1.85 (1.79, 1.91)	< 0.001	2.23 (2.15, 2.32)	< 0.001
1912–1916	1.29 (1.17, 1.42)	< 0.001	1.36 (1.26, 1.47)	< 0.001	1.91 (1.87, 1.96)	< 0.001	2.24 (2.18, 2.31)	< 0.001
1917–1921	1.23 (1.13, 1.34)	< 0.001	1.39 (1.30, 1.49)	< 0.001	2.00 (1.96, 2.04)	< 0.001	2.25 (2.20, 2.30)	< 0.001
1922–1926	1.19 (1.10, 1.28)	< 0.001	1.39 (1.31, 1.48)	< 0.001	2.01 (1.98, 2.04)	< 0.001	2.21 (2.16, 2.25)	< 0.001
1927–1931	1.17 (1.09, 1.25)	< 0.001	1.40 (1.33, 1.48)	< 0.001	2.00 (1.97, 2.03)	< 0.001	2.16 (2.12, 2.20)	< 0.001
1932–1936	1.14 (1.06, 1.21)	< 0.001	1.40 (1.33, 1.47)	< 0.001	1.94 (1.90, 1.97)	< 0.001	2.07 (2.03, 2.11)	< 0.001
1937–1941	1.11 (1.05, 1.18)	< 0.001	1.39 (1.33, 1.46)	< 0.001	1.77 (1.74, 1.80)	< 0.001	1.89 (1.85, 1.94)	< 0.001
1942–1946	1.09 (1.03, 1.15)	0.002	1.36 (1.30, 1.42)	< 0.001	1.56 (1.53, 1.59)	< 0.001	1.69 (1.64, 1.73)	< 0.001
1947–1951	1.09 (1.04, 1.15)	< 0.001	1.35 (1.30, 1.40)	< 0.001	1.35 (1.32, 1.37)	< 0.001	1.45 (1.41, 1.50)	< 0.001
1952–1956	1.06 (1.02, 1.11)	0.003	1.29 (1.24, 1.33)	< 0.001	1.18 (1.15, 1.20)	< 0.001	1.28 (1.24, 1.32)	< 0.001
1957–1961	1.04 (1.00, 1.08)	0.044	1.21 (1.18, 1.25)	< 0.001	1.04 (1.02, 1.07)	< 0.001	1.14 (1.10, 1.18)	< 0.001
1962–1966	0.98 (0.95, 1.01)	0.257	1.12 (1.09, 1.15)	< 0.001	0.93 (0.91, 0.96)	< 0.001	1.02 (0.98, 1.06)	0.343
1967–1971	0.96 (0.94, 0.99)	0.007	1.09 (1.06, 1.11)	< 0.001	0.81 (0.79, 0.84)	< 0.001	0.88 (0.84, 0.92)	< 0.001
1972–1976	0.89 (0.87, 0.91)	< 0.001	1.00 (0.98, 1.02)	0.810	0.72 (0.69, 0.74)	< 0.001	0.78 (0.74, 0.82)	< 0.001
1977–1981	0.87 (0.86, 0.89)	< 0.001	0.99 (0.97, 1.01)	0.244	0.67 (0.65, 0.70)	< 0.001	0.71 (0.67, 0.75)	< 0.001
1982–1986	0.87 (0.86, 0.88)	< 0.001	0.99 (0.97, 1.01)	0.170	0.64 (0.62, 0.66)	< 0.001	0.65 (0.62, 0.69)	< 0.001
1987–1991	0.86 (0.85, 0.87)	< 0.001	0.95 (0.94, 0.96)	< 0.001	0.59 (0.57, 0.61)	< 0.001	0.57 (0.54, 0.60)	< 0.001
1992–1996	0.78 (0.77, 0.79)	< 0.001	0.89 (0.88, 0.90)	< 0.001	0.51 (0.50, 0.53)	< 0.001	0.48 (0.45, 0.51)	< 0.001
1997–2001	0.74 (0.73, 0.75)	< 0.001	0.79 (0.78, 0.81)	< 0.001	0.49 (0.48, 0.51)	< 0.001	0.41 (0.38, 0.43)	< 0.001
2002–2006	0.69 (0.68, 0.71)	< 0.001	0.63 (0.61, 0.64)	< 0.001	0.46 (0.45, 0.48)	< 0.001	0.34 (0.32, 0.37)	< 0.001
2007–2011	0.73 (0.71, 0.75)	< 0.001	0.47 (0.46, 0.49)	< 0.001	0.45 (0.43, 0.46)	< 0.001	0.31 (0.28, 0.34)	< 0.001
2012–2016	0.80 (0.77, 0.82)	< 0.001	0.35 (0.34, 0.37)	< 0.001	0.43 (0.41, 0.45)	< 0.001	0.28 (0.25, 0.32)	< 0.001
2017–2021	0.79 (0.76, 0.82)	< 0.001	0.23 (0.21, 0.24)	< 0.001	0.39 (0.37, 0.41)	< 0.001	0.25 (0.21, 0.30)	< 0.001

RR, relative risk; CI, confidence interval.

In China, after moving the period and cohort factors, aged 0–4 years (2.99, 95% CI: 2.84, 3.16), aged 85–89 years (2.03, 95% CI: 1.92, 2.13) and aged 75–79 years (2.02, 95% CI: 1.94, 2.09) were the top 3 age groups with highest RRs of leukemia incidence. The most leukemia–related death were found for those aged 90–94 years (3.12, 95% CI: 2.95, 3.31), aged 85–89 years (3.07, 95% CI: 2.94, 3.20), and aged 80–84 years (2.39, 95% CI: 2.31, 2.48). For the period effects, the RR of incidence increased from 0.94 (95% CI: 0.93, 0.95) to 1.09 (95% CI: 1.07, 1.11), while the RR of mortality decreased from 1.11 (95% CI: 1.10, 1.13) to 0.92 (95% CI: 0.91, 0.93). In terms of cohort effects, the RRs of incidence and death were 0.79 (95% CI: 0.76, 0.82) and 0.23 (95% CI: 0.21, 0.24) in 2021, respectively.

In the US, for the age effects, aged 90–94 years (5.30, 95% CI: 5.18, 5.41), aged 85–89 years (4.70, 95% CI: 4.63, 4.78), and aged beyond 95 years (4.42, 95% CI: 4.26, 4.59) were the top 3 age groups with highest RRs of leukemia incidence. The most leukemia-related death were found in the population aged beyond 95 years (9.61, 95% CI: 9.24, 9.99), aged 90–94 years (8.55, 95% CI: 8.32, 8.78), and aged 85–89 years (6.97, 95% CI: 6.83, 7.12). For the period effects, the RR of incidence slightly increased from 0.96 (95% CI: 0.95, 0.97) to 0.99 (95% CI: 0.98, 1.00), and the RR of death increased from 0.94 (95% CI: 0.93, 0.95) to 1.05 (95% CI: 1.04, 1.06). For cohort effects, the RRs of incidence and death were respectively 0.39 (95% CI: 0.37, 0.41) and 0.25 (95% CI: 0.21, 0.30) in 2021.

The age effects showed that children and older individuals in China have higher RR for incidence, while in the US older adults in particular have higher RRs. Young children have a higher burden of incidence of leukemia in China than in the US. For the period effects, the RRs of leukemia incidence slightly increased over the past 30 years both in China and the US. The cohort effects showed the late birth cohort had lower RRs for incidence and mortality in both countries.

### Projection of incidence and mortality

**Figure 5:**
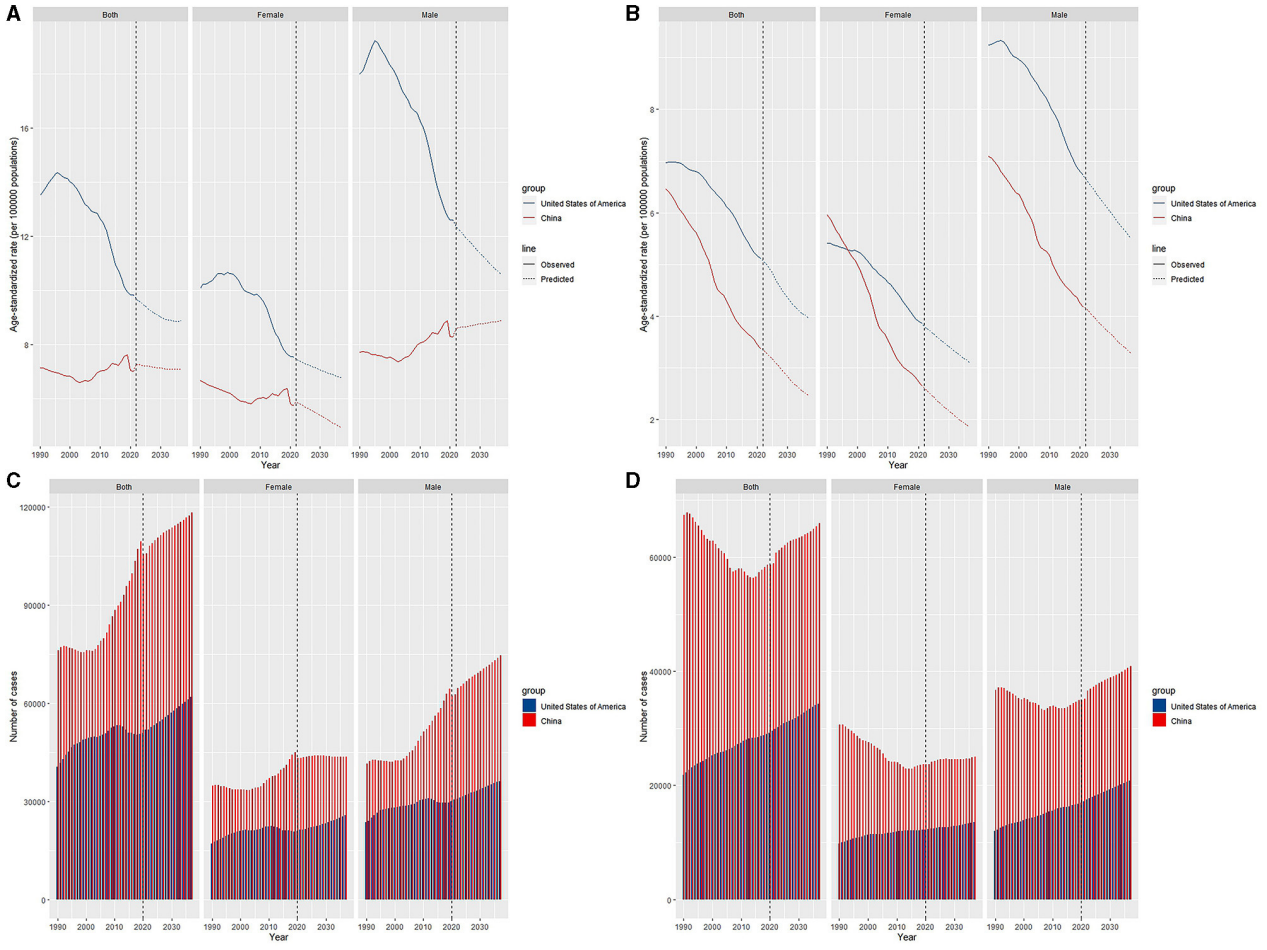
Trends and predicted results in age-standardized incidence rates **(A)**, incidence cases **(C)**, age-standardized death rates **(B)**, and death cases **(D)** of leukemia from 1990 to 2036 in China and the US.

Regardless of sex, the ASIRs of leukemia patients will maintain a downtrend over the following 15 years in the US (Figure 5). In China, the ASIR will increase in males and decrease in females, and overall, the ASIR will decrease first and then increase. In the prediction of ASDRs, two countries will show a declining trend in the next 15 years.

## Discussion

Leukemia is a severe public health issue and is a heavy burden in most areas (2). To explore these differences, our study first focused on leukemia and made a systematic and comprehensive comparison of the secular trends in incidence and mortality over the past decades between China and the US, and we made projections for the next 15 years.

The incidence burden in China has showed stable, while the analyze of mortality showed decline. Due to the policy of reformation and opening, China's economy has developed rapidly, and medical status has improved in the past 30–40 years (25), which has contributed to lower rate of death. However, at the same time, population growth, the aging process, and benzene pollution caused by large-scale industrial development may result in increasing incidence rate since 2005 (18, 26, 27). Both the incidence and mortality burdens of leukemia declined sharply in the US. While the US has a greater ASDR than China, which may be attributed to the high body mass that increases the risk of death during leukemia treatment (26, 28). The US has a greater burden of AML, which has the highest mortality rate among the subtypes (29). Because of the limitations of laboratory detection technology, other leukemia subtypes made up a proportion of leukemia subtypes in China in 1990. With the advancement of diagnostic technology, subtypes are being diagnosed more accurately (30). The diagnostic gap between the two countries is narrowing.

Males had higher leukemia ASIRs and ASDRs. Previous studies reported the same results (27, 31, 32). Tobacco, formaldehyde, benzene and obesity are thought to be associated with a higher death rate from leukemia (33, 34). Males prefer smoking (35), and they have more chances to work outdoors, which makes them contact toxic substances frequently (36). With poorer immunomodulatory function than in females (37), they may be more likely to develop leukemia than females are.

Age has the most prominent effect on leukemia incidence. Our study showed that children and older adult in China have a greater risk of incidence and mortality, while in the US, older adults have a much higher risk of incidence and mortality. That is, China had a much greater burden on the young population than did the US. This result may be due to many factors. First, infants and children are more susceptible to benzene and ionizing radiation, which have been shown to increase the risk of incidence and death (38, 39). In China, pregnant women may be at a greater risk of exposure to benzene and related compounds than US females because of unhealthy dietary habits, poorer socioeconomic circumstances, unhealthy dietary habits, and so on (40). Second, it is widely accepted that early-age leukemia is closely related to maternal smoking, paternal preconception smoking and secondhand tobacco exposure (41, 42), which cause MLL rearrangements and RAS mutations (43–45). However, in China, ~3.0% of pregnant women smoke (46), which is lower than the 7.2% reported in the US (47). Chinese men consume nearly 40% of the world's tobacco (48), accounting for 53.4% of Chinese women who are exposed to secondhand tobacco (49). Thus, more exposure to secondhand smoke and paternal preconception smoking may lead to more childhood leukemia than in the US. In China, lymphoid leukemias are the most common subgroup (50), with a peak incidence between 1 and 4 years (51), and are the most common cause of death and DALYs among children (52). A previous study revealed that East Asia had the fastest increase in the ASIR (33). In addition, the incidence of ALL in older individuals is increasing each year (51, 53). However, in the US, the most common subtype of AML (54) is AML, which is usually diagnosed in individuals older than 65 years or older (34). Differences in the proportions of subtypes were associated with differences in age. Overall, greater efforts, such as tobacco restriction and environmental protection, are urgently needed in China. Older individuals have a greater risk of incidence and mortality in both countries due to decreased immune function (55) and mutations in hematopoietic stem cells with aging (56, 57).

The period effects showed a gradual increase in incidence over time in the two countries. This can be attributed to improvements in diagnostic techniques and procedures and increased disease screening (18), deteriorating environmental pollution caused by vigorously developing the industrial economy (58), and unhealthy lifestyles (12). The period effects of death declined over time in China but increased in the US. In recent decades, China has made great progress in medical technology. Cellular immunotherapy and targeted therapy have greatly improved the survival rate of patients with leukemia (59). In the US, although medical technology has developed, the effects of economic change, such as decreasing food prices, have increased the percentage of adolescents (60, 61) and adults (62) who are morbidly obese in recent decades (63). Obesity is associated with poor outcomes (64). This may explain why USs have an increasing risk of mortality over time.

Earlier birth cohorts have higher incidence and mortality rates than later birth cohorts in China and the US. Wars such as the World War in the past may have resulted in radiation, as well as polluted food and water (65, 66), increasing the incidence and number of deaths in past eras. Luckily, with the rapid development of the internet, younger generations in both countries have more channels to obtain knowledge about disease prevention, such as taking supplements of folic acid when pregnant, which decreased the risk of leukemia in the late birth cohort (17, 67, 68). In the middle and late twentieth centuries, China and the US devoted efforts to smoking to reduce tobacco intake, resulting in reduced cigarette exposure in the late birth cohort (69, 70).

The incidence of leukemia will continue to decrease in the next 15 years in the US for both males and females. However, in China, the ASIR will increase exclusively in males, which could be caused by men are more likely to smoke and work outdoors, making them contact more carcinogenic substances and have a greater chance of developing leukemia (27). The ASDR will decline in the future in these two countries because of greater health awareness of disease, better health policies, more efficient medical technology and so on.

The limitations of our study are as follows. First, the leukemia data from GBD 2021 were reconstructed from various sources with different qualities, which may have resulted in a discrepancy with the epidemiological data (12, 16, 18). And the accuracy of result depended on the original data collected in GBD, while the deviation caused by data source quality could be inevitable (71). Second, age-period-cohort analysis could contribute to ecological fallacies by considering a community as the unit of observation and analysis. Third, although IE has the advantages of non-bias, validity, superior estimation and asymptotic properties, the essence of parameter estimation is still difficult to explain clearly due to its complex theoretical basis (72). Finally, the definition and classification of leukemia have been updated over time, and the results of our study should be interpreted prudently.

## Conclusion

Our study performed a detailed and systematic analysis of leukemia in China and the US. Over the past decades, the ASDRs in two countries both tended to decrease. And compared to the US, China had lower leukemia incidence and mortality. However, the ASIRs in China tended toward stable, which was showed downtrend in the US. Among all subtypes, the incidence of CLL has increased in China, and ALL has the heaviest disease burden. In the US, AML showed an stable trend and made its burden most. Age, period, and cohort effects had certain impacts on the leukemia burden in the two countries. China has a much greater burden on children with leukemia than the US. In the next 15 years, the incidence burden will continue to decrease, and the gap will narrow further. In short, efforts such as creating a better environment and improving medical techniques should be made to reduce the burden of leukemia.

## Data availability statement

Publicly available datasets were analyzed in this study. This data can be found at: https://vizhub.healthdata.org/gbd-results/.

## Author contributions

XG: Writing – original draft, Writing – review & editing. YTa: Writing – review & editing. LZ: Writing – review & editing. QZ: Writing – review & editing. JF: Writing – review & editing. LY: Writing – review & editing. YTo: Writing – review & editing. SZ: Writing – review & editing.
